# Phytoestrogen Biological Actions on Mammalian Reproductive System and Cancer Growth

**DOI:** 10.3797/scipharm.1007-15

**Published:** 2010-12-31

**Authors:** E Zhao, Qing Mu

**Affiliations:** 1 Department of Biology, University of Ottawa, Gendron Hall, 30 Marie Curie, K1N 6N5, Ottawa, ON, Canada; 2 School of Pharmacy, Fudan University, 826 Zhangheng Road, 201203, Pudong, Shanghai, China

**Keywords:** Phytoestrogen, Estrogen Receptor, Breast Cancer, Ovarian Cancer

## Abstract

Phytoestrogens are a family of diverse polyphenolic compounds derived from nature plant that structurally or functionally mimic circulating estrogen in the mammalian reproductive system. They induce estrogenic and anti-estrogenic effects in the brain-pituitary-gonad axis (a principal endocrine system involving in reproductive regulation) and peripheral reproductive organs. The dichotomy of phytoestrogen-mediated actions elucidates that they play the biological activities via complex mechanisms and belong to various chemical classes. In comparison with their unobvious physiological functions in normal reproductive tissues, there are increasing investigations showing that phytoestrogen induces significant inhibitory effects on the growth of breast and ovarian cancers through different signaling pathways. This review summarized the results of the previous studies regarding principal signaling transductions for mediating the growth of the ovarian and breast cancers. Phytoestrogen potentially modulates the signaling molecules via: (1) blocking the nuclear and membrane estrogen receptors (ER), (2) interfering with the growth factor receptor, (3) inhibiting the G protein-coupled receptor in ER-deficient cells, (4) activating apoptosis and nullifying anti-apoptotic signals.

## Introduction

**Fig. 1.**
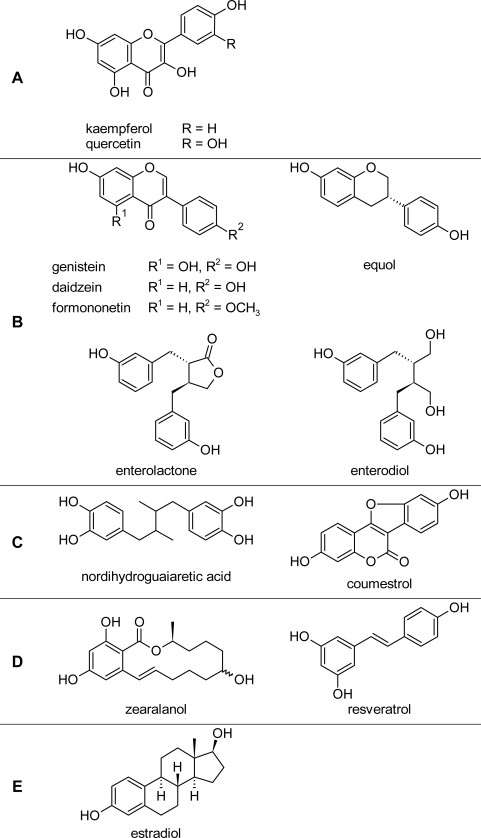
The structures of various phytoestrogens.

Phytoestrogen refers a group of polyphenolic molecules originating from exogenous nature dietary plant source, including several family members such as flavones (Kaempferol and quercitin) (Fig. 1A), isoflavones (genistein, daidzein, formononetin and equol) (Fig. 1B), lignans (enterolactone, enterodiol and nordihydroguaiaretic acid), coumestanes (coumestrol) (Fig. 1C), mycotoxins (zearalenol) and stilbens (resveratrol) (Fig. 1D) [1]. Several plants are dietary consumption of human and animals, such as, soys and beans, containing high levels of various phytoestrogens which exhibit hormone-like functions in the mammalian reproductive system [2]. The mechanism of the phytoestrogen bioactivity might be the possible binding to estrogen receptors (ERs) because of its structural similarity with estradiol (E2, 17β-estradiol) (Fig. 1E). The binding affinity to ERs is determined by a planar ring system, two ring structures divided by two carbon atoms, and spacing between hydrophobic and hydrogen bond interaction [3]. Although most of phytoestrogens have the weaker ER-binding affinities than natural estrogens, they play diverse significant estrogenic or anti-estrogenic roles on the regulation of reproduction-related organs [4, 5]. The classic biological effect of E2 is mediated through two distinct intracellular receptors, ERα and ERβ. ERα is a major subtype in the tissues of breast, uterus, cervix, vagina and other reproductive organs. ERβpimarily distributes in the ovary, prostate, testis, spleen, lung, hypothalamus and thymus [6]. Phytoestrogen exhibits a low affinity for binding to ERα suggesting their weak estrogenic activities (10^−2^–10^−3^-fold) in comparison with E2, whereas they also exert partial anti-estrogenic effect because of relatively preferentially binding to ERβ [1, 7]. The dichotomy of classic ER modulating action induced by phytoestrogen probably provides an explanation on conflicting evidence about the risks and benefits of dietary phytoestrogens in research presenting a contradictory picture in the mammalian reproductive system.

Phytoestrogen is demonstrated to exert various biological activities that are not mediated by activating nuclear ER [8, 9]. In human aortic smooth muscle cells, phytoestrogen showed an inhibitory effect on mitogen-induced proliferation, migration and extracellular matrix synthesis via down-regulating the signaling pathway of mitogen-activated protein kinases (MAPK) [10]. Quercetin, a flavone, exerted a dual effect on regulating the activities of the protein kinases in Ehrlich ascites tumor cells through increasing the cyclic AMP (cAMP)-dependent and decreasing the cAMP-independent protein kinase activities [11]. The ER-dependent and ER-independent effect of phytoestrogen indicate multiple mechanisms directing potential physiological and/or pharmacological actions in reproductive system and relevant diseases [12]. Herein, we will focus on considering the multiple effects of these phytoestrogen molecules on circulating estrogen-regulated endocrine systems which are involved in controlling mammalian reproduction. Furthermore, we will summarize the biological activities of phytoestrogen on various signaling pathways in the mammalian breast and ovarian cancer cells.

## Discussion

### Phytoestrogen targets in reproductive system

#### Phytoestrogen actions in the brain-pituitary-gonad axis

The brain-pituitary-gonad axis is the principal endocrine system to regulate mammalian reproduction. In this axis, reproductive regulation by the brain involves a complex interaction of different endocrine factors controlling pituitary gonadotropin hormone release, such as gonadotropin releasing hormone (GnRH), dopamine and γ-aminobutyric acid. In mammals, gonadotropin hormones are classified as follicle stimulating hormone (FSH) and luteinizing hormone (LH). Both FSH and LH are released into the general circulation to stimulate sex steroid production, including those of E2 and testosterone (T), and to control ovulation in females or sperm generation in males. On the other hand, E2 not merely modulates the function of reproductive organs, e.g. breast, ovary and testis, but also has positive and negative feedback roles on gonadotropin or its upstream hormone synthesis and secretion, through direct interaction with ER [13]. Therefore, similar to the structure of E2 [3], phytoestrogen plays a role in mimicking or antagonizing the E2 function. This point is worth considering in this system.

In the brain, GnRH is known and named for its function as the final common hypothalamic signal peptide to regulate reproduction. It is reported that GnRH is modulated by phytoestrogen. The flavanone 8-prenylnaringenin, identified as a potent phytoestrogen and an estrogenic compound, exhibited an E2-mimic negative feedback to inhibit the activity of the hypothalamic GnRH pulse generator and thereby serum LH levels in rat [14]. Coumestrol, another kind of phytoestrogen, exerted a direct inhibitory effect on GnRH gene expression in the GnRH neuron, and then elicited an inhibitory regulation on the reproductive system via binding to ERβ [15]. However, another investigation demonstrated that coumestrol inhibited GnRH effects on LH release by antagonizing the neuroendocrine action of estrogen thru ERα, suggesting that the estrogenic activity of phytoestrogen is mediated via different mechanisms in the brain [16].

Considering phytoestrogen modulating gonaodotropin generation and secretion in the pituitary, the previous investigations exhibited some contradictory patterns. Whereas both the GnRH-inhibited effect of coumestrol and selective endocrine activity of genistein (an isoflavone) led to reducing LH release at the level of rat pituitary [17, 18], high-dose administration of dietary equol (another isoflavone metabolite) markedly enhanced serum LH level in the ovariectomized rat [19], and intracerebroventricular of ganister increased the percentage of LHβ-subunit gene expressing cells in the anoestrous ewe pituitary via activation of cellular ERα [20]. FSH is another kind of gonadotropin released from the pituitary. Genistein had no effect either on FSH cellular immunoreactivity or on the FSHβ gene expression possibly due to no ERα existence on FSH-gonadotrophs in ewe [20]. Dichotomal phenomenon of phytoestrogen on gonadotropin secretion has been also detected in a clinical study of postmenopausal women: mean LH release decreased after discontinuing dietary isoflavone intake, but one of these women was observed to have a higher LH secretion after treatment [21]. However, recent evidence that a high-dose and long-term treatment of dietary puerarian mirica (an herb containing phytoestrogen) might produce a clear reduction in the monkey urinary FSH levels, suggested the potential regulatory effect of phytoestrogen on FSH release [22]. The results obtained from clinical and experimental studied on the regulation of gonadotropin indicates the complex regulatory mechanism of phytoestrogen activities in the pituitary, and further studies are required to explore the signaling pathways in this modulation after phytoestrogen activation.

Under the pituitary regulation, ovary is the female gonad organ that secretes steroid hormone and yields oocytes. Several lines of studies on steroidogenesis indicated the effects of phytoestrogen effects might be mediated indirectly via gonadotropin regulation or directly in the ovary. Previous results demonstrated that the treatments of dietary isoflavones will lead to a decrease, an increase or a lack of variation in serum progesterone (P4) and E2 levels. This fact suggested several inconsistent patterns of phytoestrogen regulatory abilities in ovarian granulosa cells [23]. Several investigations reported that the direct effect of phytoestrogen was related to modulation of steroidogenic enzymes in the steroid production process. Hydroxysteroid dehydrogenase/isomerase (3β-HSD) is a key enzyme to catalyze P4 production. Isoflavones inhibited the 3β-HSD enzyme activity via the post-cAMP pathway to reduce P4 generation in porcine ovary [24]. In the mammalian E2 synthesis, aromatase is an essential enzyme to aromatize T (released from theca cells) for producing E2 in ovarian granulose cells. It was noted that flavones and isoflavones dose-dependently inhibited of the generation of aromatase in human granulose-luteal cells [25, 26]. In addition, phytoestrogen was shown to play a critical role to control oocyte generation. Genistein exposure was able to inhibit oocyte nest breakdown and attenuate oocyte cell death during mouse oocyte development [27], and to decrease P4 and E2 concentration in mouse ovarian differentiation [28].

The male testis contains the reproductive part and the endocrine part that play roles in sperm-forming and sex hormone (T) production, respectively. The previous research on phytoestrogen showed these compounds had various actions on steroidogenesis. In adult male rat, exposure to high-dose coumestrol has been demonstrated to reduce T levels, possibly mediated by ERβ [29]. In rat testicular Leyding cells, isoflavone decreased the cellular production of steroid hormone but increased serum levels of T under a high-dose and long-time treatment [30]. In comparison with the phytoestrogen effect on the oocyte generation, the administration of dietary phytoestrogen at a high level in male rats blocked spermatogenesis and induced germ cell apoptosis, possibly due to disruption of estrogenic regulation in testis [31]. Another line of evidence indicated simultaneous exposure to phytoestrogen and other endocrine disrupting compounds led to deterioration of male rat testis [32].

#### Phytoestrogen actions in peripheral reproductive organs (prostate and breast)

Prostate is a complex tubuloalveolar exocrine gland in the male mammalian reproductive system. There are few investigations concerning the effect of phytoestrogen on normal prostate. A histological study of calf prostate tissues did not show that phytoestrogen diet increased basal cell proliferation but elongated the basal cells in most animals [33]. Another study indicated that only a long-term exposure of dietary genistein induced a visible histological change in rat male prostate [34]. These two investigations indicated that phytoestrogen could not induce any significant modification in the normal mammalian prostate. However, phytoestrogen intake might offer protective effects against prostate cancer in clinical study [35]. The experimental studies on the mechanisms of its inhibitory activities against prostate cancer revealed that:

○ genistein showed an ability to avoid prostate cells from oxidative stress-related DNA damage that accumulated progressively with age and led to prostate cancer [36];
○ enterolactone, a major metabolite of plant-based lignans, induced loss of mitochondrial membrane potential, release of cytochrome C and production of caspase-3 in human prostate carcinoma, suggesting that a mitochondrial-mediated, caspase-dependent apoptosis pathway was activated to inhibit tumor cell proliferation [37];
○ phytoestrogen led to an obvious increment of the ERβ generation, and ERβ is an essential factor to suppress the protstate tumor growth [38, 39].

Breast glands are the important female peripheral organs for producing and secreting milk. Regarding phytoestrogen effects on this organ, the dietary genistein exposure could alter mammary development in neonatal mice [40]. Phytoestrogen has been reported to play a critical role in the protection against breast tumors, and the mechanisms of their activities in mammary cancer cells have been widely investigated, which we will consider minutely in the next chapter.

### Mechanisms of phytoestrogen effects on various reproductive cancers

Recent studies in clinic indicated that dietary soy protein affected the growth of various reproductive cancers, such as breast and ovarian cancers [41–43]. Regarding the mechanisms of phytoestrogen effects on these cancers, the chemical structure of phytoestrogen resembles that of E2 suggesting that ER-activated genomic and/or non-genomic signaling pathways might mediate principal function of phytoestrogen [3]. Moreover, other micro-environmental regulatory molecules regulated by phytoestrogen are also considered in these reproductive cancers.

#### Phytoestrogen protection against breast cancer

Breast cancer is a kind of common malignancies with the high mortality rate in Western population. However, the people in the Asian societies have much lower incidences of this cancer than the individuals from the Western societies. Several investigations have been performed to explain this phenomenon, for instance, the Japanese women had markedly high concentrations of circulating and excreting phytoestrogen, indicating that the low mortality in breast cancer might be due to high soy intake [44, 45]. Moreover, a case-control study conducted among Asian-American women in Los Angeles County showed that soy intake reduced breast cancer risk, confirming the inverse association between dietary phytoestrogen and breast cancer [46]. Although phytoestrogen potentially exhibited an obvious protective effect in the breast cancer therapy in clinical studies, further basic biological investigations are necessary for obtaining theoretical support in the further experimental research. Herein, the mechanism of phytoestrogen action on integrating signaling pathways in breast cancer will be discussed for providing a molecular basis of future pharmacological studies of these compounds.

**Fig. 2.**
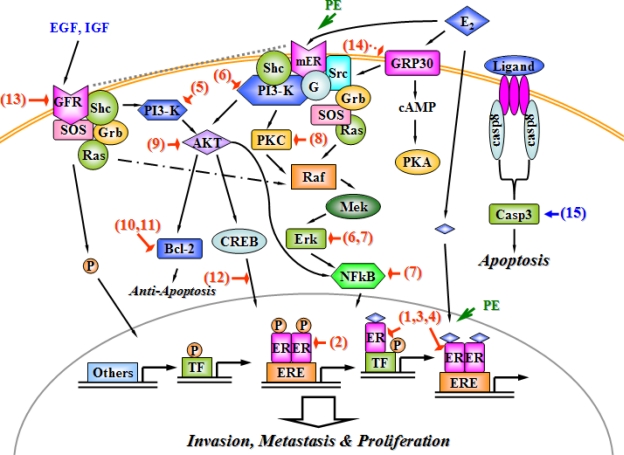
A dynamic model of phytoestrogen actions on modulating signaling pathways in the breast cancer cell. The arrows and hammers respectively present stimulation and inhibition. Abbreviations: E2, estradiol; PE, phytoestrogen; ER, estrogen receptor, mER; membrane estrogen receptor; ERE, estrogen response element; TF, transcription factor; P, phosphorylation; EGF, epidermal growth factor; IGF, insulin-like growth factor; GFR, growth factor receptor; SOS, son of sevenless; Grb, growth factor-bound protein; Ras, related RAS viral oncogene homolog; PI3-K, phosphatidylinositol 3-OH kinase; cAMP, cyclic adenosine 3′,5′-monophosphate; CREB, cAMP response element binding protein; PKC, protein kinase C; Raf, v-raf-1 murine leukemian viral oncogene; Mek, mitogen/extracellular signal protein kinase; NFkB, nuclear factor-kappaB; PKA, protein kinase A; casp8, caspase-8; casp3, caspase-3.

The signaling pathways mediating the apoptosis, invasion, metastasis and proliferation of breast cancer cells have been simplified and described in Fig. 2, which potentially mediate the phytoestrogen effects on breast cancer. They are composed of nuclear ER (genomic ER)-initiated, membrane ER (non-genomic ER)-mediated, growth factor (GF)-transduction, G protein receptor (GPR)-directed, and apoptotic signaling pathways. The activated nuclear ERα and/or ERβ could induce the modification of gene expression in breast cancer. Although encoded by unique genes, ERα and ERβ have certain functional domains with a highly similar affinity to ligand- and DNA-binding sites, suggesting that these two receptors might play redundant roles. The mammary cell has a predominant distribution of ERα [6, 47]. In the classic pathway to activate transcriptomes, the activated ERs dimerizes and forms a complex for binding to a specific DNA sequence, the estrogen response element (ERE). In non-classic pathway, E2-ER complex interacts with several transcription factors such as AP1, SP1 and NFkB to modulate gene expression without ERE [48]. Another genomic pathway is mediate by a ligand-independent manner, showing that GF activates intracellular kinase pathway to phosphorylate ER at ERE-containing promoters [6]. Apart form nuclear genomic action, the membrane ER has been demonstrated as a G protein-coupled receptor. The activated membrane ER induces the discretion of the G-protein α subunit to trigger the activation of Src kinases and PI3 kinase (PI3-K), respectively followed by downstream protein kinase C (PKC) and kinase cascade to extra-cellular regulated protein kinase (Erk), which are involved in proliferation and survival of breast cancer cells [47]. The PI3-K activation also triggers the recruitment of AKT, a serine-threnine kinase to control cell survival via activating downstream anti-apoptotic signals (e.g. Bcl-2) and transcription factors (e.g. NFkB and CREB) [49, 50]. In addition, growth factor receptors (GFR), including insulin-like growth factor receptor (IGFR) and epidermal growth factor receptor (EGFR), are the other E2-membrane signaling pathways within breast cancer cells. IGFR mediates the signal pathway through tethering ERα to plasma membrane, activating EGFR, and initiating PI3-K and Erk signaling [51]. In the ER-negative breast cancer cell, E2 directly interacted with GPR30 to induce the activation of downstream Scr-coupled Erk and cAMP/PKA signaling networks via Gβg-subunit protein [49]. The apoptotic signaling pathway is inactivated in most cancer cells, including a group of proteases such as caspase-8 and caspase-3 that cross-talk with EGF survival signaling network [50].

**Fig. 3.**
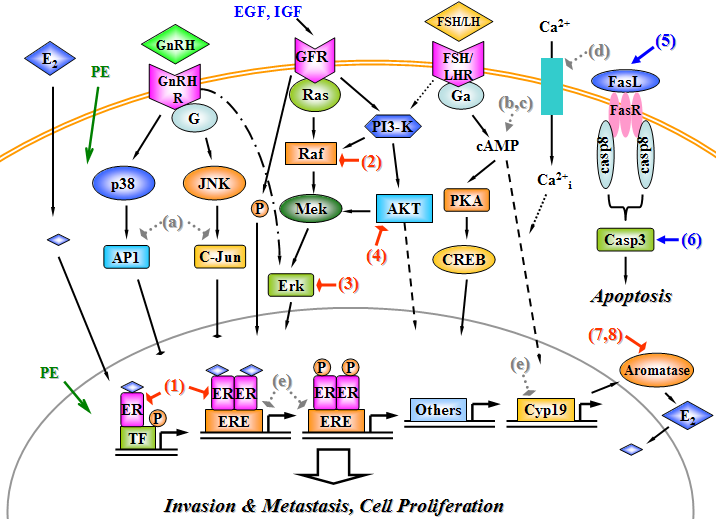
A dynamic model of phytoestrogen actions on modulating signaling pathways in the ovarian cancer cell. Stimulation and inhibition are respectively indicated as arrows and hammers. Abbreviations: E2, estradiol; PE, phytoestrogen; ER, estrogen receptor; ERE, estrogen response element; TF, transcription factor; P, phosphorylation; GnRH, gonadotropin releasing hormone; GnRHR, GnRH receptor; FSH, follicle stimulating hormone; LH, luteinizing hormone; FSH/LHR, FSH and/or LH receptor; EGF, epidermal growth factor; IGF, insulin-like growth factor; GFR, growth factor receptor; PI3-K, phosphatidylinositol 3-OH kinase; cAMP, cyclic adenosine 3′,5′-monophosphate; CREB, cAMP response element binding protein; Raf, v-raf-1 murine leukemian viral oncogene; Mek, mitogen/extracellular signal protein kinase; PKA, protein kinase A; FasL, Fas ligand; casp8, caspase-8; casp3, caspase-3; JNK, c-jun N-terminal kinase; AP1, activator protein-1; Ca^2+^, calcium ion; Ca^2+^_i_, intracellular Ca^2+^.

The intracellular mechanisms of phytoestrogen protection against cellular proliferation of breast cancers might be via: (1) binding to nuclear ER and inhibiting genomic ER-mediated gene expression, (2) interaction with membrane ER, blocking protein kinases and suppressing transcription factors, (3) inhibiting GFR activation and its downstream signaling networks, (4) activating caspases to initiate cellular apoptosis, (5) reducing the G-protein mediated signaling pathway in the ER-negative mammary cancer cell [red hammers and blue arrows in Fig. 2]. The nuclear ER interaction is the most completely studied mechanism of phytoestrogen effect. The phytoestrogen-rich pueraria mirifica showed a strong competitive binding ability to ERα and/or possibly synthesized suppressor of ERα in the therapy of rat mammary tumor [52, Fig. 2-(1)]. Phytoestrogen is recently demonstrated to modulate ERα through the activation of ligand-independent pathway. Ginsenoside, a phytoestrogenic ingredient extracted from ginseng root, preferentially activated ERα via the phosphorylation of a transcription factor (AF-1) without the ligand-receptor interaction [53, Fig. 2-(2)]. However, isoflavone was demonstrated to selectively activate ERβ rather than ERα in the breast cancer cell line MCF-7, indicating a potential ERβ-relevant mechanism underlying protective effect of dietary phytoestrogen against mammary tumor [54, Fig. 2-(3)]. Moreover, genistein (an isoflavone) might induce a self-limiting mechanism of E2-stimulated ERβ gene expression in breast cancer cells [55, Fig. 2-(4)].

Although individual phytoestrogen interacted with various types of ER, most of phytoestrogen effects were ERα-dependent due to the predominant expression of this subtype in breast cells [6]. Furthermore, 8-prenylnaringenin was proven to activate membrane ERα and to interfere with an ER associated PI3-K pathway, resulting in the apoptosis and proliferation of MCF-7 breast cells [56, Fig. 2-(5)]. In the same cell line, resveratrol was also demonstrated to inactivate non-genomic ERα-mediated PI3-K/PKC signaling network because of lacking of a specific chemical structure part in this chemical. Meanwhile, resveratrol interfered with the Src-activated MAPK (including Mek and Erk) pathway, to reduce the generation of transcription factors [57, Fig. 2-(6)]. In ER-deficient mammary tumor cells, isoflavone selectively inhibited nuclear NFkB transduction of specific target gene through a new mechanism via depressing upstream Erk and Mek activities [58, Fig. 2-(7)]. Several other investigations have demonstrated the phytoestrogenic modulation of the PI3-K downstream signaling pathway. Genistein offspring not only elevated the quantity of ER binding sites, but also markedly repressed PKC activity in carcinogen-induced mammary tumorigenesis of female rats, [59, Fig. 2-(8)]. Regarding their regulatory effect on another downstream signaling, AKT, phytoestrogen stimulated the protein expression of the tumor suppressor PTEN that led to inhibiting the function of the AKT kinase in the growth of MCF-7 breast cancer cells [60, Fig. 2-(9)]. Bcl-2 is an anti-apoptotic protein controlled by AKT kinase; genistein showed an inhibitory effect on the Bcl-2 phosphorylation in human breast cells [61, Fig. 2-(10)]. Licochalcone-A, a flavone-like molecule, exerted an anti-tumor cytotoxic effect on MCF-7 cells in a manner of repressing the anti-apoptotic Bcl-2 and modifying the bcl-2/bax ratio to induce apoptosis [62, Fig. 2-(11)]. Regarding CREB (a signaling factor in the AKT pathway), isoflavone was capable of attenuating the interaction between the kinase and its promoters to decrease the cyclooxygenase-2 expression. The cyclooxgenase-2 is an important factor to mediate the breast carcinogenesis [63, Fig. 2-(12)].

Besides modifying ER signaling networks, phytoestrogen, including genistein, exhibited a stimulatory action on the EGFR expression but did not trigger the activation of downstream signaling target (Mek and Erk) in the mammary gland, indicating a potential ER-independent mechanism underlying their protection against breast cancer [64, Fig. 2-(13)]. In the ER-negative breast cancer cells, two major kinds of phytoestrogens, genistein and quercetin, modulated the activation of growth factors via the G protein-coupled receptor homologue GPR30. The competitive binding of phytoestrogens and natural E2 to GRP30 were speculated as a new implication for understanding phytoestrogenic protection against breast cancer progression [65, Fig. 2-(14)]. Based on the finding of caspase-3 deficiency in MCF-7 cells, genistein exerted a significantly strong anti-tumor effect with the reconstitution of caspase-3, suggesting that a caspase-dependent pathway initiated by phytoestrogens to induce the apoptosis of breast cancer cells [66, Fig. 2-(15)]. Taken together, phytoestrogen plays an important role in modulating mechanisms of ER-dependent and ER-independent signaling pathways, and would be recognized as a useful therapy for breast cancers in future clinical studies.

#### Phytoestrogen effects on ovarian cancer

Ovarian cancer is the most lethal disease affecting woman healthy, which is the sixth most common cancer and the fifth leading cause of cancer-related death in Western societies [67]. In comparison with the research in breast cancers, phytoestrogens were less considered and investigated in the therapy of ovarian cancers in human and other mammalian models. Until 1997, the first study on phytoestrogenic protection against the ovarian cancer was demonstrated that genistein exerted a synergetic action with quercetin to decrease proliferation of human ovarian carcinoma OVCAR-5 cells *in vitro* [68]. The following investigation detected the inhibitory actions of the isoflavone family against the ovarian cancer, including genistein and daidzein. These two isoflavones independently down-regulated the growth of two ovarian cancer cell lines, Caov-3 and NIH: OVCAR-3, *in vitro* [69]. Furthermore, an *in vivo* research concerning the 7,12-dimethylbenz[*a*]anthracene (DMBA)-induced rat ovarian carcinogenesis confirmed the anti-tumor activity of genistein *in vitro* [70]. In addition, a recent clinical report described that a woman with platinum-refractory ovarian cancer entered into a phase of prolonged disease stabilization after a long-term intake of a soy beverage which contained rich of isoflavones, providing a strong support that phytoestrogens exerted a potential inhibitory effect on the human ovarian cancer [71]. These facts from the *in vitro*, *in vivo* and clinical research indicate that phytoestrogen might play a potential role to suppress the invasion, metastasis and growth of ovarian cancers.

The molecular aspects of phytoestrogen effects on the growth and survival of ovarian cancer cells mainly include nuclear ER-regulated gene expression, GnRH receptor (GnRHR)-, FSH and/or LH receptors (FSHR/LHR)- and GFR-mediated signal transduction, and apoptotic signaling pathway, which are described in Fig. 3. ERβ but not ERα exhibits a primary expression in the ovary; and its function might be mostly modulated by nuclear ER-mediated signaling, including classical ligand-dependent, ligand-independent and ERE-independent manner [6]. Under the regulation of gonadotropin hormone secreted from the pituitary, ovarian cancers show the high expression of FSHR and LHR on the cellular membrane. The interaction between gonadotropin and FSH/LHR is considered to induce the proliferation of some ovarian cells, via the activation of the G-protein α subunit that rapidly increases the intracellular cAMP concentration. The cyclic AMP subsequently activates the downstream PKA. However, it is still unclear whether the action of FSH/LHR is also mediated by the ERK and/or PI3-K/AKT signaling pathways in ovarian cancer cells [67; 72]. Moreover, the activation of PI3-K and the phosphorylation of consequent AKT are the key signaling events for IGF interaction with GFR, providing a potential link with FSH/LHR-mediated signaling transduction in ovarian granulose cells. Moreover, GFR activation is also capable of initiating the Erk signaling cascade [72]. GnRH and its receptor are distributed in most of human ovarian epithelial tumors, showing antiproliferative activity via the activation of G protein and its following MAPK signal cascades (such as JNK). Furthermore, GnRH-activated receptor (GnRHR) induces the activation of p38 kinase and AP-1, and decreases the Erk function to inhibit proliferation of ovarian cancer cells [67]. The inhibition of the Fas/Fas ligand (FasL) apoptotic system maybe determines the growth of ovarian tumor. Regarding FasL-associated signaling transduction, FasL might interact with Fas receptor on the cell membrane to form a Fas-DD complex. This complex activates caspase-8 protease, which recruits caspase-3 to an active form for the further induction of apoptosis [73].

With regard to phytoestrogenic functions on modulating intracellular signals to affect the ovarian cancer growth [red hammers and blue arrows in Fig. 3], the previous research on the isoflavones showed that genistein and daidzein were capable of altering cytokine (interleukin-6) synthesis and attenuating ovarian cancer cell proliferation through activating the nuclear ER-dependent pathway [69, Fig. 3-(1)]. In EGFR-coupled signaling transduction of human ovarian tumor, genistein reduced the generation of Raf and its downstream signal molecules *in vivo* and *in vitro* [57, Fig. 3-(2)]. Furthermore, pretreatment of a soy bean trypsin inhibitor suppressed the transforming growth factor-mediated activation of the Erk kinase; Erk is a major factors to mediate ovarian cancer cell invasion [74, Figure 3-(3)]. In addition, genistein decreased the level of phosphorylated AKT kinase, leading to apoptosis of ovarian cancer cells [75, Fig. 3-(4)]. Concerning other regulations of cellular apoptosis, genistein induced the gene expression of Fas (an apoptotic ligand), and suppressed bcl-2 transcription (an anti-apoptotic signal) in ovarian tumor-implanted nude mice [76, Fig. 3-(5)]. Moreover, caspase-3 function was activated by various doses of genistein treatments, suggesting another mechanism of anti-tumor effects for phytoestrogen to induce apoptotic pathway in the ovarian tumor growth [77, Fig. 3-(6)]. It is evident that several isoflavones inhibited aromatase, the enzyme that aromatize T to E2, in human ovarian granulose cells. Genistein, biochanin A and daidzein exhibited the inhibitory activities on the aromatase production *in vitro*, indicating that the phytoestrogen-induced aromatase suppression contributes to reducing the intracellular E2 production and subsequently inhibiting hormone-dependent ovarian cancer growth in an indirect manner [25, 26, Fig. 3-(7, 8)].

Although phytoestrogen might modify several signal transductions to affect the ovarian tumor cell proliferation, there is remarkably less work in ovarian cancer than the widespread studies of phytoestrogens in breast cancer. How these compounds influence other major receptors (e.g. gonadotropin receptor and GnRHR) mediated signal cascades in the ovarian cancer cell is a key question to be clarified in the future investigations. Herein, in this paragraph, we will consider some potential regulatory effects of phytoestrogen, which are indicated as grey hatch hammers or arrows in Fig. 3; these activities have been demonstrated in other relevant vertebrate cells. With regard to GnRHR-activated signaling, the expressions and activities of c-Jun and AP1 transcription factors were abolished under the administration of the phytoestrogen α-zearalanol in human umbilical vein endothelial cells [78, Fig. 3-(a)]. Some certain phytoestrogens might modulate the FSH/LHR-mediated signaling pathways. Niculescu and her/his colleagues demonstrated that dietary isoflavones could induce the translation of various genes in human lymphocytes, including those encoding cAMP-activated and G protein-coupled signaling molecules [79, Fig. 3-(b)]. Moreover, the other finding confirmed that a nongenomic action of genistein contributed to activation of cAMP/PKA signaling system in intact bovine aortic endothelial cells and human umbilical vein endothelial cells [80, Fig. 3-(c)]. Furthermore, genistein attenuated the calcium ion (Ca^2+^) entry into fura-2-loaded human platelets, implying another role of phytoestrogens on the regulation of the cellular cAMP signaling system [81, Fig. 3-(d)]. On the transcriptional level inside the teleost cell nuclear, phytoestrogen was demonstrated to alter the gene expressions of Cyp 19 (gene encoding aromatase), cAMP responsive elements, ERE and half-EREs. It hinted a new cue to investigate potential phytoestrogen modulation on gene translation and transcription in mammalian cells, especially the ovarian cancer cell [82, Fig. 3-(e)].

## Conclusion

Most current investigations showed the dichotomal phenomenon and/or unobvious pattern of phytoestrogen influence on the reproductive system. Thus, it is speculated that phytoestrogen might not exhibit any significant toxicity or side-effect on the healthy reproductive organs, which needs to be clarified in the future. Furthermore, there has been a great development in understanding the mechanisms of the phytoestrogenic effects. These mechanisms mediated via diverse signaling pathways concerning proliferation of reproductive cancers are inside or outside cellular nuclear, ER-dependent or ER-independent, and apoptotic or anti-apoptotic. Based on these previous evidences of phytoestrogen regulatory effects considered in this review, it is intriguing to deduce that phytoestrogens may have several remarkably pharmacological functions in the therapies of various hormone-dependent reproductive tumors in clinic. Consequently, the long-term research of phytoestrogens in the reproductive system will focus on determining the precise mechanisms of these phytoestrogen-associated compounds in the common malignant diseases (such as ovarian, prostate and uterus cancers), establishing the pharmacological and pharmacokinetic profiles for the individual phytoestrogen and/or its efficient extractions, and strengthening clinical studies on population or patients with or without dietary soy consumption.

## References

[1] Benassayag C, Perrot-Applanat M, Ferre F (2002). Phytoestrogens as modulators of steroid action in target cells. J Chromatogr B Analyt Technol Biomed Life Sci.

[2] Mazur W (1998). Phytoestrogen content in foods. Baillieres Clin Endocrinol Metab.

[3] Turner JV, Agatonovic-Kustrin S, Glass BD (2007). Molecular aspects of phytoestrogen selective binding at estrogen receptors. J Pharm Sci.

[4] Kuiper GG, Lemmen JG, Carlsson B, Corton JC, Safe SH, van der Saag PT, van der Burg B, Gustafsson JA (1998). Interaction of estrogenic chemicals and phytoestrogens with estrogen receptor beta. Endocrinology.

[5] Korach KS (1994). Insights from the study of animals lacking functional estrogen receptor. Science.

[6] Hall JM, Couse JF, Korach KS (2001). The multifaceted mechanisms of estradiol and estrogen receptor signaling. J Biol Chem.

[7] Tuohy PG (2003). Soy infant formula and phytoestrogens. J Paediatr Child Health.

[8] Cos P, De Bruyne T, Apers S, Vanden Berghe D, Pieters L, Vlietinck AJ (2003). Phytoestrogens: recent developments. Planta Med.

[9] Limer JL, Parkes AT, Speirs V (2006). Differential response to phytoestrogens in endocrine sensitive and resistant breast cancer cells in vitro. Int J Cancer.

[10] Dubey RK, Gillespie DG, Imthurn B, Rosselli M, Jackson EK, Keller PJ (1999). Phytoestrogens inhibit growth and MAP kinase activity in human aortic smooth muscle cells. Hypertension.

[11] Graziani Y, Chayoth R, Karny N, Feldman B, Levy J (1982). Regulation of protein kinases activity by quercetin in Ehrlich ascites tumor cells. Biochim Biophys Acta.

[12] Cassidy A, Faughnan M (2000). Phyto-oestrogens through the life cycle. Proc Nutr Soc.

[13] Crowley WR (1999). Toward Multifactorial Hypothalamic Regulation of Anterior Pituitary Hormone Secretion. News Physiol Sci.

[14] Christoffel J, Rimoldi G, Wuttke W (2006). Effects of 8-prenylnaringenin on the hypothalamo-pituitary-uterine axis in rats after 3-month treatment. J Endocrinol.

[15] Bowe J, Li XF, Sugden D, Katzenellenbogen JA, Katzenellenbogen BS, O’Byrne KT (2003). The effects of the phytoestrogen, coumestrol, on gonadotropin-releasing hormone (GnRH) mRNA expression in GT1-7 GnRH neurones. J Neuroendocrinol.

[16] Jacob DA, Temple JL, Patisaul HB, Young LJ, Rissman EF (2001). Coumestrol antagonizes neuroendocrine actions of estrogen via the estrogen receptor alpha. Exp Biol Med (Maywood).

[17] McGarvey C, Cates PA, Brooks A, Swanson IA, Milligan SR, Coen CW, O’Byrne KT (2001). Phytoestrogens and gonadotropin-releasing hormone pulse generator activity and pituitary luteinizing hormone release in the rat. Endocrinology.

[18] Hughes CL, Chakinala MM, Reece SG, Miller RN, Schomberg DW, Basham KB (1991). Acute and subacute effects of naturally occurring estrogens on luteinizing hormone secretion in the ovariectomized rat: Part 2. Reprod Toxicol.

[19] Rachoń D, Vortherms T, Seidlová-Wuttke D, Wuttke W (2007). Effects of dietary equol on the pituitary of the ovariectomized rats. Horm Metab Res.

[20] Polkowska J, Ridderstråle Y, Wańkowska M, Romanowicz K, Misztal T, Madej A (2004). Effects of intracerebroventricular infusion of genistein on gonadotrophin subunit mRNA and immunoreactivity of gonadotrophins and oestrogen receptor-alpha in the pituitary cells of the anoestrous ewe. J Chem Neuroanat.

[21] Nicholls J, Lasley BL, Nakajima ST, Setchell KD, Schneeman BO (2002). Effects of soy consumption on gonadotropin secretion and acute pituitary responses to gonadotropin-releasing hormone in women. J Nutr.

[22] Trisomboon H, Malaivijitnond S, Cherdshewasart W, Watanabe G, Taya K (2007). Assessment of urinary gonadotropin and steroid hormone profiles of female cynomolgus monkeys after treatment with Pueraria mirifica. J Reprod Dev.

[23] Dusza L, Ciereszko R, Skarzyński DJ, Nogowski L, Opałka M, Kamińska B, Nynca A, Kraszewska O, Słomczyńska M, Woclawek-Potocka I, Korzekwa A, Pruszyńska-Oszmałek E, Szkudelska K (2006). Mechanism of phytoestrogens action in reproductive processes of mammals and birds. Reprod Biol.

[24] Tiemann U, Schneider F, Vanselow J, Tomek W (2007). In vitro exposure of porcine granulosa cells to the phytoestrogens genistein and daidzein: Effects on the biosynthesis of reproductive steroid hormones. Reprod Toxicol.

[25] Lacey M, Bohday J, Fonseka SM, Ullah AI, Whitehead SA (2005). Dose-response effects of phytoestrogens on the activity and expression of 3beta-hydroxysteroid dehydrogenase and aromatase in human granulosa-luteal cells. J Steroid Biochem Mol Biol.

[26] Rice S, Mason HD, Whitehead SA (2006). Phytoestrogens and their low dose combinations inhibit mRNA expression and activity of aromatase in human granulosa-luteal cells. J Steroid Biochem Mol Biol.

[27] Jefferson W, Newbold R, Padilla-Banks E, Pepling M (2006). Neonatal genistein treatment alters ovarian differentiation in the mouse: inhibition of oocyte nest breakdown and increased oocyte survival. Biol Reprod.

[28] Chen Y, Jefferson WN, Newbold RR, Padilla-Banks E, Pepling ME (2007). Estradiol, progesterone, and genistein inhibit oocyte nest breakdown and primordial follicle assembly in the neonatal mouse ovary in vitro and in vivo. Endocrinology.

[29] Tarragó-Castellanos CR, García-Lorenzana CM, Diaz-Sánchez V, Velázquez-Moctezuma J (2006). Gonadotrophin levels and morphological testicular features in rats after different doses of the phytoestrogen coumestrol. Neuro Endocrinol Lett.

[30] Akingbemi BT, Braden TD, Kemppainen BW, Hancock KD, Sherrill JD, Cook SJ, He X, Supko JG (2007). Exposure to phytoestrogens in the perinatal period affects androgen secretion by testicular Leydig cells in the adult rat. Endocrinology.

[31] Assinder S, Davis R, Fenwick M, Glover A (2007). Adult-only exposure of male rats to a diet of high phytoestrogen content increases apoptosis of meiotic and post-meiotic germ cells. Reproduction.

[32] Kilian E, Delport R, Bornman MS, de Jager C (2007). Simultaneous exposure to low concentrations of dichlorodiphenyltrichloroethane, deltamethrin, nonylphenol and phytoestrogens has negative effects on the reproductive parameters in male Spraque-Dawley rats. Andrologia.

[33] Groot MJ (2006). Effects of phyto-oestrogens on veal calf prostate histology. Vet Res Commun.

[34] Michael McClain R, Wolz E, Davidovich A, Pfannkuch F, Edwards JA, Bausch J (2006). Acute, subchronic and chronic safety studies with genistein in rats. Food Chem Toxicol.

[35] Martin JH, Crotty S, Nelson PN (2007). Phytoestrogens: perpetrators or protectors. Future Oncol.

[36] Raschke M, Rowland IR, Magee PJ, Pool-Zobel BL (2006). Genistein protects prostate cells against hydrogen peroxide-induced DNA damage and induces expression of genes involved in the defence against oxidative stress. Carcinogenesis.

[37] Chen LH, Fang J, Li H, Demark-Wahnefried W, Lin X (2007). Enterolactone induces apoptosis in human prostate carcinoma LNCaP cells via a mitochondrial-mediated, caspase-dependent pathway. Mol Cancer Ther.

[38] Stettner M, Kaulfuss S, Burfeind P, Schweyer S, Strauss A, Ringert RH, Thelen P (2007). The relevance of estrogen receptor-beta expression to the antiproliferative effects observed with histone deacetylase inhibitors and phytoestrogens in prostate cancer treatment. Mol Cancer Ther.

[39] Hedelin M, Bälter KA, Chang ET, Bellocco R, Klint A, Johansson JE, Wiklund F, Thellenberg-Karlsson C, Adami HO, Grönberg H (2006). Dietary intake of phytoestrogens, estrogen receptor-beta polymorphisms and the risk of prostate cancer. Prostate.

[40] Jefferson WN, Padilla-Banks E, Newbold RR (2007). Disruption of the developing female reproductive system by phytoestrogens: genistein as an example. Mol Nutr Food Res.

[41] Ha TC, Lyons-Wall PM, Moore DE, Tattam BN, Boyages J, Ung OA, Taylor RJ (2006). Phytoestrogens and indicators of breast cancer prognosis. Nutr Cancer.

[42] McCann SE, Freudenheim JL, Marshall JR, Graham S (2003). Risk of human ovarian cancer is related to dietary intake of selected nutrients, phytochemicals and food groups. J Nutr.

[43] Rannikko A, Petas A, Raivio T, Jänne OA, Rannikko S, Adlercreutz H (2006). The effects of short-term oral phytoestrogen supplementation on the hypothalamic-pituitary-testicular axis in prostate cancer patients. Prostate.

[44] Morton MS, Arisaka O, Miyake N, Morgan LD, Evans BA (2002). Phytoestrogen concentrations in serum from Japanese men and women over forty years of age. J Nutr.

[45] Adlercreutz H, Honjo H, Higashi A, Fotsis T, Hämäläinen E, Hasegawa T, Okada H (1991). Urinary excretion of lignans and isoflavonoid phytoestrogens in Japanese men and women consuming a traditional Japanese diet. Am J Clin Nutr.

[46] Wu AH, Yu MC, Tseng CC, Twaddle NC, Doerge DR (2004). Plasma isoflavone levels versus self-reported soy isoflavone levels in Asian-American women in Los Angeles County. Carcinogenesis.

[47] Levin ER, Pietras RJ (2008). Estrogen receptors outside the nucleus in breast cancer. Breast Cancer Res Treat.

[48] Yamaguchi Y (2007). Microenvironmental Regulation of Estrogen Signals in Breast Cancer. Breast Cancer.

[49] Castoria G, Migliaccio A, D’Amato L, Di Stasio R, Ciociola A, Lombardi M, Bilancio A, Di Domenico M, de Falco A, Auricchio F (2008). Integrating signals between cAMP and MAPK pathways in breast cancer. Front Biosci.

[50] Henson ES, Gibson SB (2006). Surviving cell death through epidermal growth factor (EGF) signal transduction pathways: implications for cancer therapy. Cell Signal.

[51] Song RX, Fan P, Yue W, Chen Y, Santen RJ (2006). Role of receptor complexes in the extranuclear actions of estrogen receptor alpha in breast cancer. Endocr Relat Cancer.

[52] Cherdshewasart W, Panriansaen R, Picha P (2007). Pretreatment with phytoestrogen-rich plant decreases breast tumor incidence and exhibits lower profile of mammary ERalpha and ERbeta. Maturitas.

[53] Lau WS, Chan RY, Guo DA, Wong MS (2008). Ginsenoside Rg1 exerts estrogen-like activities via ligand-independent activation of ERalpha pathway. J Steroid Biochem Mol Biol.

[54] Chrzan BG, Bradford PG (2007). Phytoestrogens activate estrogen receptor beta1 and estrogenic responses in human breast and bone cancer cell lines. Mol Nutr Food Res.

[55] Cappelletti V, Miodini P, Di Fronzo G, Daidone MG (2006). Modulation of estrogen receptor-beta isoforms by phytoestrogens in breast cancer cells. Int J Oncol.

[56] Brunelli E, Minassi A, Appendino G, Moro L (2007). 8-Prenylnaringenin, inhibits estrogen receptor-alpha mediated cell growth and induces apoptosis in MCF-7 breast cancer cells. J Steroid Biochem Mol Biol.

[57] Li Y, Mi C, Wu YZ, Yang SF, Yang ZQ (2004). [The effects of genistein on epidermal growth factor receptor mediated signal transduction pathway in human ovarian carcinoma cells lines SKOV3 and its xenograft in nude mice]. Zhonghua Bing Li Xue Za Zhi.

[58] Vanden Berghe W, Dijsselbloem N, Vermeulen L, Ndlovu N, Boone E, Haegeman G (2006). Attenuation of mitogen- and stress-activated protein kinase-1-driven nuclear factor-kappaB gene expression by soy isoflavones does not require estrogenic activity. Cancer Res.

[59] Hilakivi-Clarke L, Cho E, Onojafe I, Raygada M, Clarke R (1999). Maternal exposure to genistein during pregnancy increases carcinogen-induced mammary tumorigenesis in female rat offspring. Oncol Rep.

[60] Waite KA, Sinden MR, Eng C (2005). Phytoestrogen exposure elevates PTEN levels. Hum Mol Genet.

[61] Liao CH, Pan SL, Guh JH, Teng CM (2004). Genistein inversely affects tubulin-binding agent-induced apoptosis in human breast cancer cells. Biochem Pharmacol.

[62] Rafi MM, Rosen RT, Vassil A, Ho CT, Zhang H, Ghai G, Lambert G, DiPaola RS (2000). Modulation of bcl-2 and cytotoxicity by licochalcone-A, a novel estrogenic flavonoid. Anticancer Res.

[63] Lau TY, Leung LK (2006). Soya isoflavones suppress phorbol 12-myristate 13-acetate-induced COX-2 expression in MCF-7 cells. Br J Nutr.

[64] Cotroneo MS, Wang J, Fritz WA, Eltoum IE, Lamartiniere CA (2002). Genistein action in the prepubertal mammary gland in a chemoprevention model. Carcinogenesis.

[65] Maggiolini M, Vivacqua A, Fasanella G, Recchia AG, Sisci D, Pezzi V, Montanaro D, Musti AM, Picard D, Andò S (2004). The G protein-coupled receptor GPR30 mediates c-fos up-regulation by 17beta-estradiol and phytoestrogens in breast cancer cells. J Biol Chem.

[66] Yang S, Zhou Q, Yang X (2007). Caspase-3 status is a determinant of the differential responses to genistein between MDA-MB-231 and MCF-7 breast cancer cells. Biochim Biophys Acta.

[67] Leung PC, Choi JH (2007). Endocrine signaling in ovarian surface epithelium and cancer. Hum Reprod Update.

[68] Shen F, Weber G (1997). Synergistic action of quercetin and genistein in human ovarian carcinoma cells. Oncol Res.

[69] Chen X, Anderson JJ (2001). Isoflavones inhibit proliferation of ovarian cancer cells in vitro via an estrogen receptor-dependent pathway. Nutr Cancer.

[70] Tanaka T, Kohno H, Tanino M, Yanaida Y (2002). Inhibitory effects of estrogenic compounds, 4-nonylphenol and genistein, on 7,12-dimethylbenz[a]anthracene-induced ovarian carcinogenesis in rats. Ecotoxicol Environ Saf.

[71] Klein A, He X, Roche M, Mallett A, Duska L, Supko JG, Seiden MV (2006). Prolonged stabilization of platinum-resistant ovarian cancer in a single patient consuming a fermented soy therapy. Gynecol Oncol.

[72] Fuller PJ, Chu S (2004). Signalling pathways in the molecular pathogenesis of ovarian granulosa cell tumours. Trends Endocrinol Metab.

[73] Fraser M, Leung B, Jahani-Asl A, Yan X, Thompson WE, Tsang BK (2003). Chemoresistance in human ovarian cancer: the role of apoptotic regulators. Reprod Biol Endocrinol.

[74] Kobayashi H, Suzuki M, Kanayama N, Terao T (2004). A soybean Kunitz trypsin inhibitor suppresses ovarian cancer cell invasion by blocking urokinase upregulation. Clin Exp Metastasis.

[75] Gossner G, Choi M, Tan L, Fogoros S, Griffith KA, Kuenker M, Liu JR (2007). Genistein-induced apoptosis and autophagocytosis in ovarian cancer cells. Gynecol Oncol.

[76] Wang X, Xin XY, Huang YH (2006). [Regulative effect of genistein on xenografted tumor of ovarian carcinoma cell on nude mice]. Zhongguo Zhong Yao Za Zhi.

[77] Gercel-Taylor C, Feitelson AK, Taylor DD (2004). Inhibitory effect of genistein and daidzein on ovarian cancer cell growth. Anticancer Res.

[78] Duan J, Xu H, Dai S, Wang X, Wu Y, Zhang Y, Sun R, Ren J (2008). Phytoestrogen alpha-zearalanol inhibits homocysteine-induced endothelin-1 expression and oxidative stress in human umbilical vein endothelial cells. Atherosclerosis.

[79] Niculescu MD, Pop EA, Fischer LM, Zeisel SH (2007). Dietary isoflavones differentially induce gene expression changes in lymphocytes from postmenopausal women who form equol as compared with those who do not. J Nutr Biochem.

[80] Liu D, Jiang H, Grange RW (2005). Genistein activates the 3′,5′-cyclic adenosine monophosphate signaling pathway in vascular endothelial cells and protects endothelial barrier function. Endocrinology.

[81] Sargeant P, Farndale RW, Sage SO (1993). ADP- and thapsigargin-evoked Ca^2+^ entry and protein-tyrosine phosphorylation are inhibited by the tyrosine kinase inhibitors genistein and methyl-2,5-dihydroxycinnamate in fura-2-loaded human platelets. J Biol Chem.

[82] Cheshenko K, Pakdel F, Segner H, Kah O, Eggen RI (2008). Interference of endocrine disrupting chemicals with aromatase CYP19 expression or activity, and consequences for reproduction of teleost fish. Gen Comp Endocrinol.

